# Bridging Liver Transplantation in the Treatment of Intestinal Failure Associated Liver Disease in Infants—A Bridge Too Far?

**DOI:** 10.3390/children9050699

**Published:** 2022-05-10

**Authors:** Abubakar Sharif, Khalid Sharif, Darius F. Mirza, Girish L. Gupte

**Affiliations:** 1 Liver Unit (Including Small Bowel Transplantation), Birmingham Women & Children’s Hospital, Birmingham B4 6NH, UK; abubakar.sharif@nhs.net (A.S.); khalid.sharif1@nhs.net (K.S.); darius.mirza@uhb.nhs.uk (D.F.M.); 2 Liver Transplant Unit, Queen Elizabeth University Hospitals Birmingham, Birmingham B15 2TH, UK

**Keywords:** intestinal failure associated liver disease, liver transplant, small bowel transplantation, ethics, risks

## Abstract

Infants with intestinal failure associated liver disease (IFALD) requiring liver and bowel transplant have a high mortality on the transplant waiting list due to the scarcity of the size-matched donor organs. Bridging liver transplantation has been used to allow the children to grow to a reasonable size so that a combined liver and small bowel transplant could be performed in the future. We report on two children with irreversible intestinal failure (ultra-short bowel syndrome secondary to gastroschisis and microvillous inclusion disease) with IFALD who underwent bridging liver transplantation at our institution. Both patients made a good recovery from their initial surgery. One patient died 6 months following surgery from generalized sepsis, and the other patient survived in good condition to undergo a combined liver and small bowel transplant but died a few days post-transplant. In the current era of scarcity of donor organs, this raises an ethical dilemma for the team involved regarding appropriate utilisation of a scarce resource.

## 1. Introduction

Parenteral nutrition (PN) is a lifesaving treatment in children with irreversible intestinal failure (IF). The most life threatening complication of IF is intestinal failure associated liver disease (IFALD), particularly when it develops and progresses within the first year of life. Combined liver and small bowel transplantation is a well established treatment for children with irreversible intestinal failure and progressive IFALD [1]. However, there is a scarcity of appropriate size-matched liver and small bowel donors. There is a high likelihood of death in children under one year of age awaiting a size-matched organ on the transplant waiting list, especially in the United Kingdom (UK). The strategy of combined en-bloc reduced liver and small bowel transplantation adopted by our unit in the past cannot prevent waiting list mortality in infants with IFALD because of the lack of small donors (<10 kg) in the UK. We have attempted ‘bridging’ liver transplantation in two patients with intestinal failure associated liver disease as a temporizing life-saving measure to allow time for a suitable small bowel or combined liver small bowel donor to be found.

This paper will highlight the ethical issues surrounding decision-making on bridging isolated liver transplantation in patients with IFALD where organs are limited, and the short or long term outcomes are still unknown.

## 2. Materials and Methods

Two patients with intestinal failure associated liver disease were referred to our unit for transplant assessment.

Case 1

A three month old with a background of ultra-short bowel syndrome secondary to gastroschisis with concomitant jejunal atresia. The length of the small bowel was 5 cm. He had progressive IFALD and was assessed and considered a suitable candidate for a combined liver and small bowel transplantation. Biochemically, he was pancytopaenic reflecting the portal hypertension. White cell count was as low as 1.4 × 10^9^/L, platelet count in the 20 s × 10^9^/L with haemoglobin as low as 6 g/dL. The transaminases—AST and ALT—were ranging between 51-1000IU/L. At the time of assessment, prothrombin time (PT) was stable between 12–14 s.

**Figure 1:**
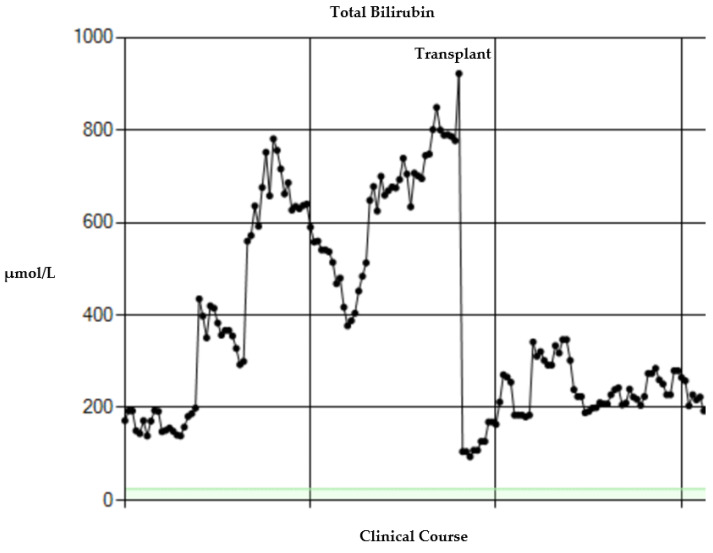
Changes in serum bilirubin before and after transplant for case 1.

Repeated episodes of generalized sepsis associated with worsening liver function tests (see Figure 1) resulted in a rapid clinical deterioration. The highest bilirubin peaked over 900 µmol/L. Following discussion with the parents, we electively decided to perform a ‘bridging’ isolated liver transplant as a life-saving procedure prior to definitive surgery. At 8 months of age, he underwent an isolated split liver transplant (segments 2 and 3). His explanted liver showed histological features of cirrhosis. He had an uneventful post-operative period and was placed on the liver and small bowel transplant list following his liver transplant. He developed recurrent episodes of generalized sepsis 3 months after isolated liver transplant, resulting in a dramatic worsening of liver function and deterioration in his general condition. He was admitted to his local paediatric intensive care unit (PICU), but despite aggressive conservative management, developed multiple organ failure and died 6 months following his isolated liver transplant.

Case 2

A 6 month old child with microvillous inclusion disease and intestinal failure associated liver disease was referred for liver and small bowel transplant assessment.

**Figure 2:**
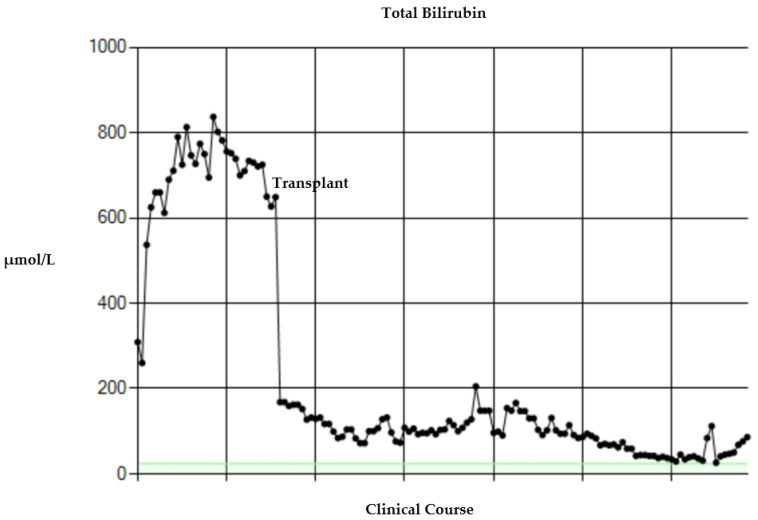
Changes in serum bilirubin before and after transplant for case 2.

This patient also had MYO5B mutation, which certainly affected the spectrum of the clinical manifestations including the cholestatic liver disease. Similar to case 1, there was biochemical evidence of portal hypertension. Platelets were low ranging from 20–80 10^9^/L prior to transplant. His serum bilirubin was increasing and rapidly rose to over 800 umol/L following a series of repeated episodes of sepsis (see Figure 2). The patient was listed for a combined liver and small bowel transplant; however, no donors were available, and his clinical condition continued to deteriorate on the transplant waiting list. After discussion with the family, he was placed on the waiting list for an isolated liver transplant as a temporizing ‘bridge’ to definitive liver and bowel transplantation at a later date. His liver biopsy, as part of his transplant assessment, revealed appearances of moderate to severe fibrosis with biliary features that fell short of established cirrhosis (fibrotic stage 4 of 6).

At the age of 11 months, he underwent an isolated split liver transplant (segments 2 and 3). There was an improvement in the liver function tests in the initial post-operative period (Figure 2). There was recurrence of IFALD in the transplanted liver at the time of liver biopsy, 15 days post-transplant. Four weeks post-transplant, he was noted to be drowsy and developed episodes of generalised and focal seizures lasting up to 20 min. CT revealed a large, acute right parieto-occipital haematoma with surrounding oedema extending into the ventricles. The neurosurgical team were involved, and via right parietal craniotomy, placed an external ventricular drain including an ICP bolt. He later developed raised intracranial pressures. A large volume of intraventricular haemorrhage was evacuated. Serial imaging revealed resolution of the haemorrhage with stable appearances of the ventricles and position of the external ventricular drain. He had made an excellent neurological recovery prior to discharge to his referring hospital. He demonstrated appropriate growth and development with good nutritional status on parenteral nutrition. He underwent a combined liver and small bowel transplant 5 months after his isolated liver transplant. This procedure was complicated by primary non-function of the graft; he deteriorated rapidly, and died three days after his combined liver and small bowel transplant.

## 3. Discussion

We have presented two cases of bridging liver transplantation. We have previously described our experiences on isolated liver transplant [2] and combined liver-intestine grafts and isolated intestinal transplantation in children [3]. Certainly due to advances in the management of patients with intestinal failure we are seeing less children being referred with severe disease.

We would like to discuss the important topics related to the ethical underpinnings of bridging liver transplantation. Following successful isolated liver transplantation, both our patients were sufficiently well to be discharged from our unit and were put on the waiting list for a combined liver and small bowel transplant. Due to lack of establishment of enteral feeds, both children developed the recurrence of IFALD in the transplanted liver. One patient died 6 months after the isolated liver transplant from multiple organ failure as a consequence of sepsis in the context of liver failure. The other patient died following a combined liver and small bowel transplant due to graft primary non-function. The ‘bridging’ liver transplant gave one of the patients the chance to undergo definitive transplantation at a later time. ‘Bridging’ liver transplant has to be differentiated from the option of isolated liver transplantation in children with short bowel syndrome (SBS) and IFALD, where criteria are well established and children can be successfully weaned from parenteral nutrition (PN) in the long-term [4].

The transplant team involved the parents in the consultation with the various members of the multidisciplinary team (MDT)—clinicians, transplant surgeons, specialist nurses, social worker, psychologist and the wider members of the MDT who were involved in the pre-, peri- and post-transplant management.

Parents were counselled in a balanced manner offering management options with an opportunity to revisit any issues at any point whilst simultaneously acknowledging the difficult nature and decisions that are being made. Parents were made aware that data on long-term outcomes following bridging liver transplant in the young infants is limited.

Ethical issues of Bridging liver transplantation

Ethics plays a huge role in medicine, especially in organ transplantation. Traditionally, transplantation has been used to prevent recipients from dying; however, we are now increasingly seeing transplants are being performed with the aim of improving the quality of life, such as hand transplantation [5].

Clearly there has been a paradigm shift from saving lives to improving the quality of life, which necessitates a shift in the ethical approach that is embedded in organ transplantation. This requires the various health care professionals and the public, including the legal team, to rethink the factors involved in saving or prolonging life with the associated risk of compromising the quality of life of others.

As organs are scarce with no current long-term data on outcomes of transplanting young infants with IFALD, it remains unclear whether bridging liver transplantation will be an accepted clinical and ethical practice in patients with severe IFALD. An ethical framework as the one outlined in Table 1 below should be used to facilitate decision-making.

The ethics surrounding counselling and managing young patients with IFALD may appear multifaceted and complex, however they are not unusual. Medicine is dynamic, and accepted practices are constantly changing. For example, up until recently, the British Association of Perinatal Medicine recommended that neonates less than 23 weeks (>22 weeks) gestation should not be resuscitated. This has now changed with the recent guidelines now recommending resuscitation of neonates >22 weeks gestation [6].

Organ transplantation presents complex ethical dilemmas to questions that do not have simple answers.

**Table 1:** Principles of medical ethics as defined by Beauchamp and Childress [7].

Principles of Ethics
Respect for autonomy—Recognising and respecting the patient’s decision-making capacity.
Beneficence—Acting in the patient’s best interest.
Nonmaleficence—Avoiding harm
Justice—A group of norms for distributing benefits, risk and costs fairly.

**Autonomy**

Informed consent is essential to ethically justify any sort of transplantation. However, an autonomous wish should never be justified on its own. Various healthcare professionals may find their personal values conflict with the decisions and opinions of colleagues. We need to accept and respect the patient’s right to autonomy. The wider MDT have an obligation not to honour every autonomous wish. Questions arise—where to draw the line when determining which patients are medically fit, how to assess donor selection, is it fair to offer organs to adult recipients with a known history of hepatitis C and substance abuse who are likely to continue post-transplant (where recurrence maybe inevitable). These questions raise moral distress and issues of fairness.

Unfortunately, national laws and the wider MDT’s decision involving ethical decisions should not merely entertain autonomous wishes. The NHSBT criteria determines that organ transplantation should be offered where there is more than 50% chance of survival >5 years [8]. The MDT team carries the responsibility of not only looking after the patient’s autonomy but also in making the right choice on behalf of the donor and donor family who have given the ‘gift of life’ in difficult circumstances.

**Beneficence**

When the MDT transplant team makes a decision about bridging liver transplantation as a lifesaving procedure, it may be acting in the patient’s best interest. Moreover, the MDT team have to consider the prospect of achieving the ultimate goal of a sequential liver/intestinal and/or intestinal graft against the balance of recurrence of IFALD in the transplanted liver with its associated risks of sepsis, rejection and opportunistic infections.

**Non-maleficence**

The MDT team may consider a bridging liver transplant in order to avoid harm to the patient and prolong life in order to get a definitive sequential bridging liver transplant. It is tempting to believe that one would not intentionally swap quantity of life for quality of life, nevertheless the MDT team should consider a careful balance between increasing the quality of life against pursuing a longer life. There are numerous moral implications for quality and quantity of life. Factors such as assessing the quality of life can be compounded by differences in individual views about acceptable levels of disability. An acceptable outcome for one individual may not be acceptable for another. Following a successful combined liver and bowel transplant, children are weaned from PN on to enteral feeds and there is an improvement in quality of life. However, children with bridging liver transplants need PN, and the burden on the family further increases as they not only continue to administer PN but also give numerous medications to support the transplant.

**Justice**

It is clear that families differ in their understanding of risk and commonly used survival figures in percentages or terms such as ‘extremely high’ or ‘high’ are used. Risk assessment needs to take into account the current parent’s knowledge, views and values. It is essential that in these difficult cases, parents are offered various options and supported to facilitate decision-making according to their needs or preferences. The risks should be conveyed sympathetically and with clarity by an experienced member of the MDT team. During the decision-making process, the MDT transplant team also has to consider the risks of death for other children with end stage liver disease on the transplant waiting list who have a 95% chance of survival as compared to the benefits of survival for the child with a bridging liver transplant. There are numerous points for discussions that the MDT team including the ethical advisory group may need to consider in reviewing such cases (Table 2).

**Table 2:** Points for discussion and ethical reflection.

Should priority be given to the sickest child or the child with the best long-term chances of benefiting? Should a liver that will have a known benefit for one child be used innovatively to help another where the chances of saving this child in the short and long term are uncertain, but where consideration has to be given to the long-term benefits of medical progress in general? Although not common practice, should exploring the role for living related donation in challenging cases be entertained?

## 4. Conclusions

Young children with acute deterioration of IFALD leading to liver failure are a challenging group of patients to consider for bridging liver transplantation. When making the final decision, the MDT transplant team should not only make a clinical assessment but should also consider ethical discussion around balancing the short- and long-term outcomes, survival and quality of life of all children on the transplant waiting list. As healthcare professionals, we have a responsibility to make sure ethically justified decisions are made.
